# A Case of *Corynebacterium diphtheriae* Native Mitral and Tricuspid Valve Infective Endocarditis Complicated by Shower Emboli

**DOI:** 10.1155/cric/8832570

**Published:** 2026-01-13

**Authors:** Sara Mashayekan, Tiffany Chow, Julia Gupta, Gary Huang

**Affiliations:** ^1^ Department of Cardiovascular Disease, Sutter Roseville Medical Center, Roseville, California, USA; ^2^ Department of Internal Medicine, UCSF Health Stanyan Hospital, San Francisco, California, USA; ^3^ Cardiovascular Medical Group of San Francisco, UCSF Health Stanyan Hospital, San Francisco, California, USA

**Keywords:** *Corynebacterium*, infective endocarditis, septic emboli

## Abstract

Infective endocarditis is a rare phenomenon that may have devastating consequences. While uncommon, the spectrum of causative organisms can include *Corynebacterium* species. *Corynebacterium diphtheriae* endocarditis bears a high potential for systemic complications and overall mortality. We present a case of mitral and tricuspid native valve endocarditis caused by *C. diphtheriae* in an immunocompetent patient and highlight the severe manifestations of this condition.

## 1. Introduction

Infective endocarditis (IE) is a severe and pervasive disease which requires rapid recognition and treatment in order to prevent poor outcomes. While commonly isolated as skin commensals [1], *Corynebacterium* species can rarely invoke fulminant systemic illnesses, including IE. The genus *Corynebacterium* encompasses a heterogeneous spectrum of catalase‐positive, gram‐positive bacteria with a number of pathogenic strains. Several species have been identified as uncommon causes of endocarditis [2], including *Corynebacterium diphtheriae*. Patients with *Corynebacterium* IE are typically afflicted by higher mortality rates as well as an increased need for surgical valve replacement as compared to IE caused by other pathogens [3].

Classical diphtheria is a highly virulent disease mediated by the hematogenous spread of diphtheria toxin [4], traditionally associated with “pseudomembrane” formation in the respiratory tract mucosa. Following the implementation of mass immunization against the diphtheria toxoid, nontoxigenic strains have risen in prominence as a source of invasive infections. While typically associated with cutaneous and respiratory infections, nontoxigenic *C diphtheriae* has been increasingly recognized as a causative agent of IE, especially as an opportunistic pathogen among populations with valvular pathology or compromised immunity. We present a case of mitral and tricuspid native valve endocarditis caused by *C. diphtheriae* in an immunocompetent patient.

## 2. Case Report

A 74‐year‐old female with no known history of prior cardiac disease presented to the emergency department with complaints of nausea, lower extremity weakness, and diffuse myalgias for 2 days. She was febrile (38.3°C) with an audible (3/6) holosystolic murmur in all four auscultatory areas of the heart. There was no evidence of skin or soft tissue infection upon examination. Shortly following her arrival, she became progressively hypotensive, with a nadir blood pressure of 86/42 mmHg, and tachycardic to a peak heart rate of 141 bpm. The patient′s preliminary blood cultures were remarkable for gram‐positive rods, for which she was started on intravenous (iv) vancomycin and piperacillin–tazobactam.

Troponin‐I level was found to be elevated to 3.48 ng/mL (normal < 0.06 ng/mL). Computed tomography (CT) angiography of the abdomen was performed, revealing multiple wedge‐shaped, hypoattenuated regions of the spleen and superior poles of both renal cortices concerning for multifocal infarctions (Figure 1a,b). CT head without contrast demonstrated acute hemorrhage throughout portions of the subarachnoid space in both the right and the left hemispheres, along with a small extra‐axial subdural hemorrhage in the left frontal lobe. Magnetic resonance imaging (MRI) without gadolinium contrast was obtained in order to better characterize her intracranial findings, confirming the presence of multiple acute bilateral supratentorial and infratentorial punctate foci of true reduced diffusion, in addition to multiple bilateral frontal vertex and posterior regions of acute subarachnoid blood. Due to concern for endocarditis, transthoracic echocardiography (TTE) was performed, demonstrating possible tricuspid and mitral vegetations; subsequent transesophageal echocardiography showed a small mitral valve vegetation with moderate‐to‐severe eccentric mitral regurgitation, in addition to a small tricuspid valve vegetation with severe tricuspid regurgitation (Figure 2a,b).

Figure 1Computed tomography of the abdomen and (a) pelvis coronal view demonstrating splenic lesions suggestive of multifocal splenic infarcts and (b) transverse view demonstrating renal cortical lesions suggestive of multifocal renal infarcts.

**Figure:**
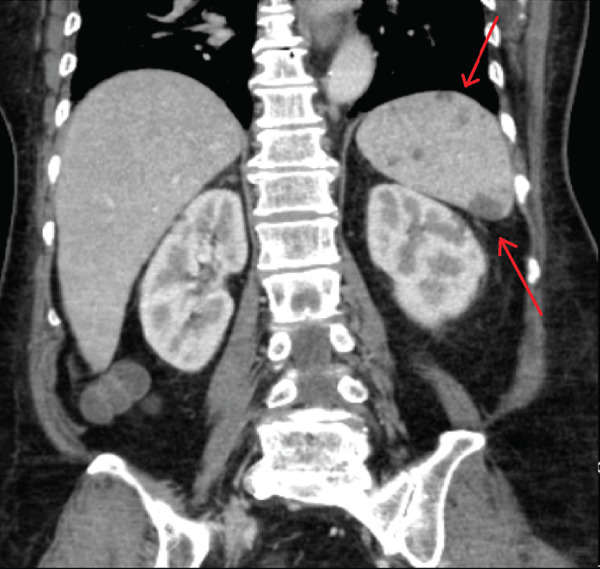
(a)

**Figure:**
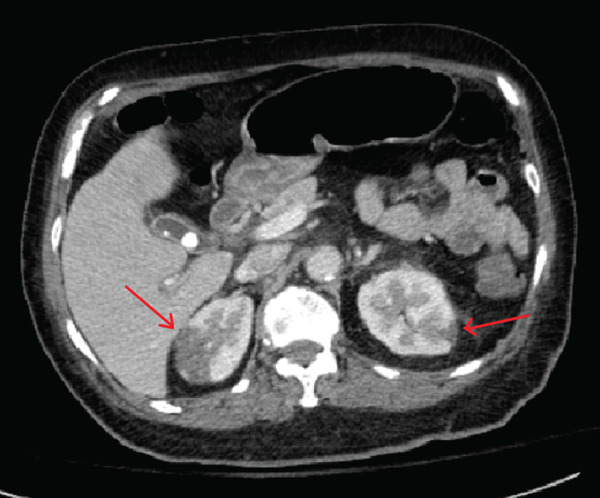
(b)

Figure 2Two‐dimensional transesophageal echocardiography demonstrating (a) a 0.7 × 0.3 cm mitral valve vegetation and (b) a 0.6 × 0.2 cm tricuspid valve vegetation.

**Figure:**
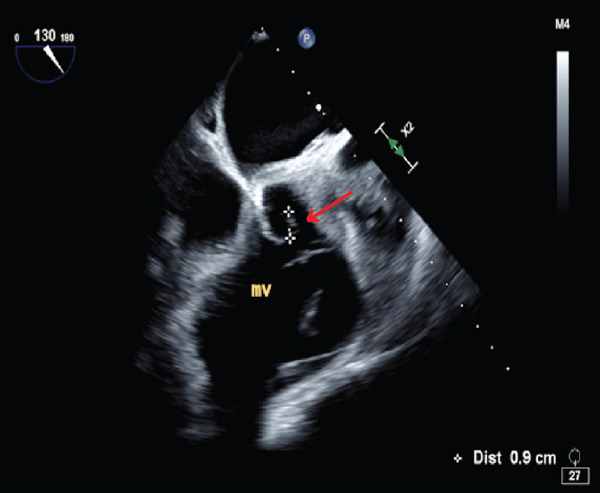
(a)

**Figure:**
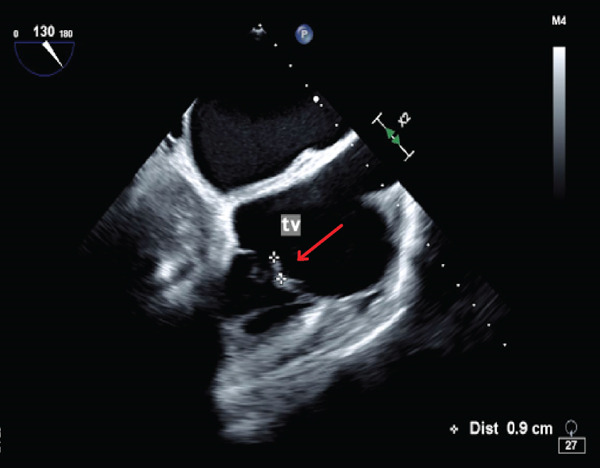
(b)

Final blood cultures revealed *C. diphtheriae* bacteremia with susceptibility to vancomycin. As it was determined that the mitral valve vegetation bore low potential for further embolic events, valvular surgery was not undertaken. Her clinical course was complicated by persistent inability to ambulate. MRI of her lumbar spine revealed severe canal stenosis at the L4‐5 level for which she underwent decompressive laminectomy and fusion. She was treated with 6 weeks of iv vancomycin. Further peripheral blood cultures were negative, and her general condition improved prior to her discharge from the hospital. As her most recent tetanus–diphtheria–acellular pertussis immunization had been administered more than a decade prior to her hospitalization, vaccination was performed in the outpatient setting. TTE was performed 3 months following discharge and demonstrated no vegetations.

## 3. Discussion

The rate of hospitalizations related to IE has experienced a steady increase over the past few decades, especially among older patient populations [5]. Risk factors include iv drug abuse, valvular heart disease, prosthetic valves, indwelling medical devices, and immunosuppressive conditions [6]. The spectrum of causative agents most commonly includes *Staphylococcus aureus*, followed by streptococci, coagulase‐negative staphylococci, enterococci, and HACEK group organisms [7]. Given the propensity of *Corynebacterium* species to colonize skin and mucous membranes, positive blood cultures are often dismissed as contaminants; however, while uncommon, cases of *Corynebacterium* IE have previously been reported. *Corynebacterium striatum* [8], *Corynebacterium jeikeium* [9], *Corynebacterium amycolatum* [10], and *C. diphtheriae* have been specifically recognized as causative strains, among others. In one review of 129 patients with *Corynebacterium* endocarditis, 43.4% of cases resulted in death [11], highlighting the malignant nature of this condition.

Both toxigenic and nontoxigenic strains of *C. diphtheriae* have demonstrated the ability to adhere to and invade endothelial cells [12], potentiating the virulence of this organism. *C. diphtheriae*, *Corynebacterium ulcerans*, and *Corynebacterium pseudotuberculosis* species possess the *tox* gene necessary for diphtheria toxoid production; the toxin exhibits robust cytotoxicity, entering respiratory mucosal cells via receptor‐mediated endocytosis to inhibit protein synthesis and induce cytolysis [4], followed by rapid dissemination of the toxin into circulation. In the absence of diphtheria toxoid, nontoxigenic *Corynebacterium* species are capable of directly adhering to and entering mucosal cells [13] by binding to fibrin, with varied degrees of efficiency. While widespread vaccination protocols have reduced the global burden of toxigenic *C. diphtheriae*, genomic variations have led to an increased prevalence of nontoxigenic *Corynebacterium* strains, especially as nosocomial pathogens.

*Corynebacterium* species have been implicated in cases of respiratory infection [14], urinary tract infections [15], postoperative osteomyelitis and septic arthritis [16], and endocarditis. *Corynebacterium* appears to have a predilection for affecting the aortic or mitral valve; prior analyses demonstrated isolated left‐sided endocarditis in 94.6% of cases of *Corynebacterium* endocarditis [12]. Among *Corynebacterium* endocarditis, *C. diphtheriae* appears to be the most prevalent species [17]. Given the rarity of *C. diphtheriae* endocarditis, the majority of published literature is limited to case studies, hindering our ability to draw broad generalizations regarding this disease. There are no current established guidelines for the specific management of *C. diphtheriae* endocarditis, though most species of *Corynebacterium* are sensitive to vancomycin [2]. Review of prior cases has shown that the majority of patients with *C. diphtheriae* endocarditis are treated with beta‐lactams alone or in combination with an additional agent [18]. To our knowledge, this is the first case of native valve *C. diphtheriae* IE with involvement of both the mitral and tricuspid valves.

The present case is remarkable both for the lack of preexisting risk factors for endocarditis and the severe manifestations of this patient′s infection, including the presence of renal, splenic, and cerebral infarcts. Multivalvular endocarditis may represent the presence of more advanced disease in this case, as evidenced by the presence of widespread emboli, reflecting the aggressive nature of *C. diphtheriae.* As with the majority of prior cases of *C. diphtheriae*, IE has been associated with at least one predisposing condition, most commonly valvular heart disease or iv drug abuse [15]. Spontaneous native endocarditis involving both the mitral and tricuspid valves appears to be an atypical presentation. Additionally, given the high prevalence of left‐sided endocarditis among patients with *Corynebacterium* endocarditis, tricuspid valve involvement is unusual. Review of prior cases of *Corynebacterium* endocarditis estimates a 34% rate of cerebrovascular accident, peripheral mycotic aneurysm, or embolism [15], highlighting the destructive nature of this pathogen. The presence of widespread septic emboli in our patient falls within the realm of expected complications. The high mortality of this condition is a testament to the high virulence of invasive *Corynebacterium*; therefore, differentiation between contaminants and true bacteremia is vital in preventing adverse outcomes.

## 4. Conclusion

*C. diphtheriae* is a rare causative agent of IE. While this infection typically involves the aortic and mitral valves, right‐sided valvular involvement remains possible, as shown in our case. In addition, due to the life‐threatening nature of this infection, *C. diphtheriae* endocarditis requires rapid recognition and treatment, and positive blood cultures should not be immediately dismissed as contaminants in affected patients.

## Ethics Statement

Ethical approval was not sought for the present case study because human or animal subject research was not performed.

## Consent

Informed consent was obtained prior to publication.

## Conflicts of Interest

The authors declare no conflicts of interest.

## Funding

No funding was received for this manuscript.

## Data Availability

Data sharing is not applicable to this article as no datasets were generated or analyzed during the current study.
